# Next-Generation Sequencing and Variant Cataloguing for Screening and Diagnosis of Mucolipidoses and Other Lysosome-Related Organelle Disorders, Including Lysosomal Membrane or Transport Disorders

**DOI:** 10.3390/genes17060643

**Published:** 2026-05-31

**Authors:** Irina Vlasova-St. Louis, Svetlana Khaiboullina

**Affiliations:** 1 Vinnana AI LLC, Sheridan, WY 82601, USA; 2 Institute of Fundamental Medicine and Biology, Kazan Federal University, Kazan 420008, Russia

**Keywords:** next-generation sequencing, lysosomal diseases, mucolipidoses types I–IV, lysosome-related organelles, whole-exome sequencing, whole-genome sequencing, Hermansky-Pudlak syndrome type I–XI, HPS

## Abstract

Next-generation sequencing (NGS) has transformed the diagnostic landscape for inherited metabolic diseases by enabling high-resolution detection of pathogenic variants across genetically heterogeneous lysosomal pathways. This is particularly impactful for lysosomal diseases (LDs), including the mucolipidoses (ML I–IV), and for disorders involving lysosomal membranes, transporters, and lysosome-related organelles (LROs). These conditions often present with overlapping biochemical and clinical features that historically complicated accurate diagnosis. This review synthesizes current knowledge on the application of next-generation sequencing (NGS) technologies in the detection and interpretation of variants underlying mucolipidoses types I-IV and selected LRO and lysosomal membrane transport disorders. We summarize expanded variant catalogues, genotype–phenotype correlations, and functional evidence informing pathogenicity classification. In addition, we discuss the integration of NGS into newborn screening and population-level genomics. Collectively, these advances have refined disease definitions, resolved diagnostically challenging cases, and reshaped clinical workflows across the LD and LRO disease spectra.

## 1. Introduction

Inherited metabolic diseases involving lysosome metabolism, storage pathways, and lysosome-related organelles encompass a broad and expanding spectrum of disorders unified by disruptions in intracellular degradation, trafficking, or membrane transport. Historically, diagnosis relied heavily on biochemical testing and clinical pattern recognition—methods that were often insufficient for diseases with extensive allelic heterogeneity, variable phenotypes, or overlapping clinical features [1,2]. Over the past decade, NGS has fundamentally reshaped this landscape by enabling the simultaneous evaluation of hundreds to thousands of gene variants, with high analytical sensitivity [3,4]. Targeted NGS panels are widely used as a cost-effective first-line approach in clinically well-defined cases [5]. Whole-exome sequencing (WES) extends analysis to coding regions and is also useful for population screening and carrier detection, particularly in populations with high rates of consanguinity [6,7,8]. Whole-genome sequencing (WGS) enables more comprehensive detection of structural variants, intronic changes, and regulatory elements that may be missed by WES [9]. In addition, RNA sequencing (RNA-seq) provides functional insight by identifying aberrant splicing, exon skipping, and mRNA transcript-level effects that cannot be reliably inferred from DNA-based approaches alone. In diagnostically challenging cases, long-read sequencing technologies, including Oxford Nanopore Technologies (ONT), further improve variant detection by resolving complex rearrangements, repetitive regions, and allele phasing. In clinical practice, a tiered diagnostic strategy is often most efficient. Targeted panels or WES with copy-number variant analysis are appropriate for patients with a clear clinical suspicion of lysosomal disease, while WGS, RNA-seq, or long-read sequencing may be more effective in cases with atypical, late-onset, or nonspecific presentations, when WES yields negative results, or when the functional impact of identified variants remains unclear. The clinical interpretation of variants identified through NGS is largely guided by standardized frameworks, most notably the American College of Medical Genetics and Genomics (ACMG) criteria, under which the majority of reported variants are classified into pathogenicity categories to support diagnostic decision-making [10].

Lysosomes and their associated organelles play a central role in cellular homeostasis by mediating macromolecular degradation, membrane turnover, and vesicular transport. Defects in lysosomal enzymes, trafficking machinery, ion channels, or membrane transporters give rise to a diverse group of nearly 90 LD and LRO disorders [3,11]. Among these, the mucolipidoses (ML I-IV) occupy a key position, representing a combination of defects in lysosomal enzyme processing, trafficking, and membrane function [12]. NGS has been transformative for LDs, including the mucolipidoses (ML I-IV), where multiple genes (e.g., *NEU1*, *GNPTAB*, *GNPTG*, *MCOLN1*) converge on shared cellular pathways but produce markedly variable phenotypes. The discovery of novel missense, nonsense, frameshifts, splice-altering, and structural variants through large-scale sequencing has refined genotype–phenotype correlations and expanded recognized disease boundaries. Beyond classical LDs, NGS has elucidated the molecular basis of disorders involving lysosomal membranes and transporters (e.g., *CTNS*, *SCARB2*, *SLC17A5*) and biogenesis defects affecting lysosome-related organelles such as melanosomes and platelet dense granules (e.g., HPS genes, which are briefly overviewed below). Many of these disorders lie at the interface between LDs and organelle biogenesis defects, reinforcing the importance of NGS in gene-disease pathogenicity assessments, reducing diagnostic odysseys, updating variant catalogues and clarification of variants’ pathogenicity, and implications for clinical diagnostics, newborn screening, and precision medicine initiatives.

## 2. Mucolipidoses

MLs are a group of rare, autosomal recessive lysosomal diseases characterized by impaired lysosomal enzyme function, processing, or intracellular trafficking. As a consequence, undegraded lipids, glycoproteins, and oligosaccharides accumulate within lysosomes in multiple tissues, leading to progressive multisystem pathology. Based on their molecular etiology, four classical forms are recognized: ML I (sialidosis), ML II (I-cell disease), ML III (pseudo-Hurler polydystrophy), and ML IV (MCOLN1-related disease) [13,14,15].

Although mucolipidoses share several overlapping clinical features (among themselves and with other LDs), including coarse facial features, dysostosis multiplex, joint stiffness, psychomotor delay, and organomegaly, the severity, onset, and clinical course vary widely. This variability largely reflects the degree of residual lysosomal enzyme activity and the molecular nature of the underlying pathogenic variants. The introduction of NGS has substantially expanded the known pathogenic variant spectrum of *NEU1*, *GNPTAB*, *GNPTG*, and *MCOLN1*, enabling the identification of numerous previously unrecognized alleles, including intronic splice-altering variants, deep intronic variants, copy-number changes, and population-specific founder mutations. Comprehensive molecular studies have revealed clear genotype–phenotype correlations across many ML subtypes and underscored the critical role of NGS-based molecular confirmation for accurate diagnosis, subclassification, and genetic counseling [16].

### 2.1. Mucolipidosis Type I (ICD-11: 5C71.3)

#### 2.1.1. Pathophysiology and Clinical Features

**Figure 1:**
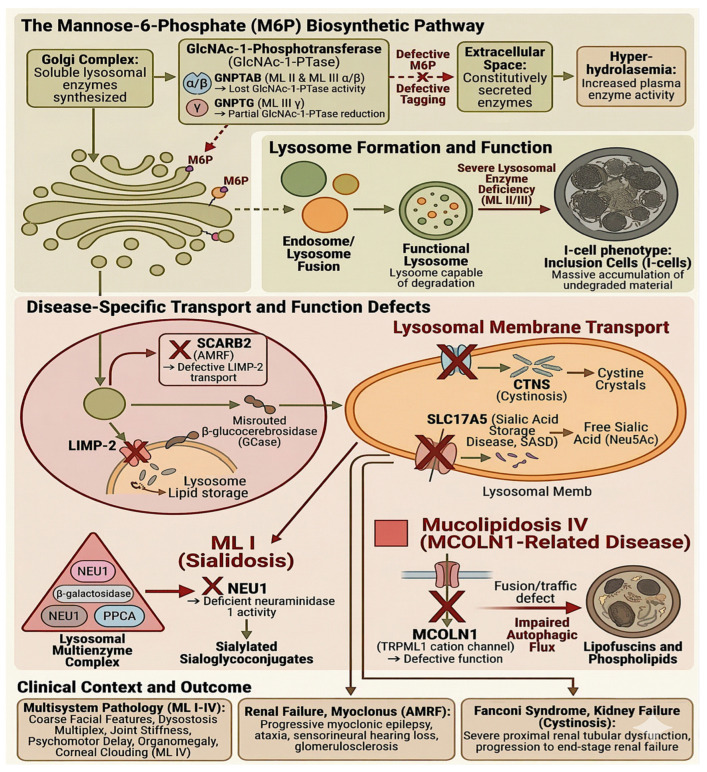
Schematic representation of intracellular trafficking of lysosomal components and associated disease pathways. This figure is designed with Gemini 2.0.

ML I, also known as sialidosis, is caused by deficient activity of neuraminidase 1 (NEU1), a lysosomal exoglycosidase responsible for removing terminal sialic acid residues from sialoglycoconjugates (Figure 1). NEU1 protein functions as part of a lysosomal multienzyme complex that includes β-galactosidase and protective protein/cathepsin A (PPCA), which is essential for NEU1 stability and catalytic activity [17]. Loss of NEU1 activity destabilizes this complex and results in the intralysosomal accumulation of sialylated glycoproteins and oligosaccharides, leading to widespread cellular dysfunction. Beyond its canonical lysosomal role, NEU1 has emerged as a regulator of neuroimmune homeostasis. Recent studies demonstrate that NEU1 modulates microglial activation through regulation of TREM2 stability and signaling, implicating NEU1 deficiency in neuroinflammatory and neurodegenerative processes observed in sialidosis [18].

Clinically, sialidosis is classified into two major phenotypic subtypes:

Type I (late-onset sialidosis): Typically presents in adolescence or adulthood with progressive myoclonus, generalized seizures, ataxia, cherry-red macular spots, and relatively preserved early neurodevelopment [13,19,20].
Type II (early-onset sialidosis): Presents in infancy or childhood with coarse facial features, hepatosplenomegaly, dysostosis multiplex, developmental delay, and early-onset neurodegeneration, often with a rapidly progressive course [21]. The differential diagnostic significance of ocular manifestations, particularly cherry-red macular spots as a pathognomonic finding for ML I or ML II, remains debated, as these findings have been reported inconsistently across both type I and type II ML phenotypes [20,21].

#### 2.1.2. Pathogenic Variants in *NEU1*

Pathogenic variants in *NEU1* include missense, nonsense, frameshift, splice-site, and copy-number alterations, most commonly occurring in compound heterozygous configurations (ClinVar_NEU1). Disease severity correlates strongly with residual neuraminidase activity, with levels above ~15% consistently associated with attenuated, late-onset phenotypes, while null or functionally null variants result in severe early-onset disease [22,23,24]. Isolated type I sialidosis is most frequently associated with compound heterozygous hypomorphic missense variants that preserve partial enzymatic activity. Recurrent variants linked to this phenotype include p.Ser182Gly, p.Arg294Cys, p.Gly277Arg, p.Pro80Leu, p.Val143Glu, p.Asp177Val, p.His337Arg, p.Arg280Gln, p.Ser233Arg, p.Asp135Asn, p.Tyr268Cys, as well as p.Tyr186Cys and p.Arg267Trp, observed across multiple cohorts and ethnic backgrounds. These genotypes are typically associated with adolescent or adult onset, progressive myoclonus, seizures, ataxia, and variable or absent ocular involvement [19,20,22,24,25,26]. Homozygous hypomorphic missense variants, and rarely, intronic variants (e.g., c.1021+4A>T) may also result in type I disease, particularly in consanguineous families [27]. Variants such as p.Asp135Asn, p.Arg294Cys and p.Arg305Pro have been identified in homozygous states and are associated with non-neuronopathic or attenuated phenotypes when residual enzymatic activity of the protein is preserved. Population-specific enrichment of certain alleles has been documented, most notably p.Ser182Gly in East Asian populations, and p.Asp135Asn in Turkish families, consistent with founder effects influencing regional mutational spectra [26,28,29]. Variants disrupting catalytic or structurally critical domains of NEU1 protein are associated with severe early-onset type II sialidosis, including nonsynonymous changes. Examples include p.Arg341Gly and p.Tyr370Cys, which directly impair catalytic function and behave as functionally null alleles. Severe infantile phenotypes are also consistently associated with truncating variants, including nonsense and frameshift mutations such as p.Arg206Ter, p.Arg341Ter, p.Glu209SerfsTer94, and p.Pro316fs, as well as multi-exon deletions, reflecting complete loss of enzyme activity [21,23,24,26,30]. The structural organization of the NEU1 protein and the distribution of pathogenic variants within the *NEU1* gene, together with their functional and phenotypic consequences, have been comprehensively characterized in prior studies [31], providing established frameworks for genotype–phenotype interpretation.

No approved enzyme replacement therapy or gene therapy is available yet for sialidoses, underscoring the importance of comprehensive NGS-based approaches for accurate diagnosis, prognosis, and genetic counseling.

### 2.2. Mucolipidosis II (ICD-11: 5C71.0)

#### 2.2.1. Pathophysiology and Clinical Presentation

ML II is a rare, severe, and typically fatal lysosomal trafficking disorder caused by pathogenic variants in *GNPTAB*, which encodes the α/β precursor subunits of N-acetylglucosamine-1-phosphotransferase (GlcNAc-1-PTase) (Figure 1). This enzyme catalyzes the first step in the synthesis of the mannose-6-phosphate (M6P) signal required for proper lysosomal targeting of acid hydrolases. Near-complete loss of GlcNAc-1-PTase activity results in misrouting and extracellular secretion of lysosomal enzymes, leading to secondary lysosomal deficiency and intracellular accumulation of undegraded substrates, accompanied by elevated plasma lysosomal enzyme activities [32,33]. Clinically, ML II presents in early infancy with severe skeletal dysplasia, coarse facial features, gingival hypertrophy, progressive joint contractures, cardiopulmonary disease, and profound global developmental arrest. Disease progression is rapid, and prognosis is poor, with most affected individuals dying in early childhood [34]. ML II represents the severe end of the GNPTAB-related disease spectrum, which also includes ML IIIα/β and intermediate phenotypes. Because clinical overlap between these forms is substantial, molecular diagnosis using NGS is essential and represents the first-line diagnostic approach [35].

#### 2.2.2. Pathogenic Variants in *GNPTAB*

GNPTAB encodes the precursor of α/β subunits, essential for the Golgi localization and catalytic activity of GlcNAc-1-PTase. Classical ML II is most commonly caused by biallelic truncating variants, including nonsense, frameshift, and canonical splice-site mutations, which abolish enzyme activity. Recurrent truncating variants associated with severe infantile phenotypes include p.Gln104Ter, p.Trp894Ter, p.Ser1058Ter, p.Arg1189Ter, p.Arg225Ter, p.Arg364Ter, p.Arg587Ter, and frameshift variants such as p.His1158fsTer15, p.Leu1168ProfsTer5, p.Lys898SerfsTer13 observed across multiple ethnic cohorts [33,36,37,38]. Recent cohort studies further demonstrate that combinations of truncating and splice-site variants consistently result in classical ML II. Examples include compound heterozygotes p.Gly508AspfsTer39 and c.3335+5G>A, p.Thr30HisfsTer24 and Lys850AsnfsTer10, p.Cys528Arg and c.2715+1G>A, p.Lys545SerfsTer16 and c.2715+1G>A, p.Thr1032HisfsTer11 and c.571+4A>G, and homozygous p.Gln802Ter, all of which are associated with severe early-onset disease [39]. Several novel pathogenic splice site variants c.3335+5G>A, c.571+4A>G, c.1284+1G>A, and c.1284+1G>T have been discovered by WGS in these studies [39,40]. The splicing mutation c.2715+1G>A has been identified as one of the most frequent pathogenic variants in GNPTAB, particularly in the Chinese population [41].

The frameshift variant p.Leu1168fs represents one of the most frequently reported *GNPTAB* pathogenic alleles worldwide and has been identified in both homozygous and compound heterozygous states in multiple populations [42,43,44]. Owing to its high prevalence and consistent association with classical ML II, this variant has become a widely used reference allele in preclinical gene therapy studies [45]. The missense p.Lys4Gln variant has been identified in compound heterozygous states with truncating alleles, including p.Leu655Serfs and p.Phe730fs, and is associated with intermediate ML II/III phenotypes reflecting partial preservation of GlcNAc-1-PTase activity [43,46]. Mechanistically, N-terminal missense variants impair Golgi retention of GlcNAc-1-PTase, leading to its aberrant lysosomal degradation rather than complete loss of synthesis. Additional conserved missense variants, including p.Ala387Val, p.Arg364Cys, and p.Arg986Ser have been reported in patients with residual enzymatic activity and intermediate disease severity; however, the full phenotype depends on the pathogenicity of the variant on the other allele [33,37,47,48]. Large deletions, duplications, and complex rearrangements involving *GNPTAB* represent an important but often underrecognized cause of ML II. Increasing evidence from curated databases (e.g., ClinVar_GNPTAB) highlights a broad spectrum of structural and complex variants, including intragenic duplications affecting splice regions (e.g., c.3250-10_3335+112dup, c.3250-16_3335+104dup), complex indels such as c.780_895delinsTTTTTATTATGGGAT (p.Leu260fs), as well as large genomic rearrangements and copy-number changes, including multi-exon deletions and duplications (e.g., NC_000012.11:g.(102155542_102157979) (102190541_102224336)del and NC_000012.11:g.(?102190445)(102190550?)dup), all contributing to disease pathogenesis [49]. Earlier NGS studies of Brazilian patients (clinically and biochemically diagnosed with ML II or III α/β) identified six novel mutations: p.Asp76Gly, p.Ser385Leu, p.Gln278LysfsTer3, p.His588GlnfsTer27, p.Asn642LeufsTer10, and p.Tyr1111Ter, allowing this cohort to be stratified by genotype [50]. These findings are supported by functional studies demonstrating that missense mutations in *GNPTAB* can impair GlcNAc-1-PTase enzyme activity through distinct mechanisms, including protein mislocalization and defective processing, thereby contributing to disease severity and phenotypic variability [50].

Rare cases of dual genetic pathology have been reported, such as combined *GNPTAB* frameshift variants (e.g., c.732_733delAA) with a pathogenic variant in an unrelated gene (*NDUFA12*, p.Arg60Ter), resulting in unusually severe infantile phenotypes [51]. Hematopoietic stem cell transplantation (HSCT) has been explored experimentally in ML II, with limited case-based evidence suggesting potential modest prolongation of survival or partial stabilization of disease progression. However, due to small sample sizes, heterogeneous protocols, and short follow-up durations, the therapeutic benefit of HSCT in ML II remains uncertain, and controlled longitudinal studies are required before clinical recommendations can be made [37].

### 2.3. Mucolipidosis III (ICD-11: 5C71.1)

#### 2.3.1. Pathophysiology and Clinical Presentation

ML III results from a partial deficiency of N-acetylglucosamine-1-phosphotransferase (GlcNAc-1-PTase), leading to reduced, but not absent, mannose-6-phosphate (M6P) tagging of lysosomal hydrolases and preservation of residual lysosomal targeting (Figure 1). This partial enzymatic activity underlies the attenuated clinical course compared with ML II. Disease onset typically occurs between early childhood and adolescence and is characterized by progressive joint stiffness and pain, short stature, mild coarsening of facial features, valvular heart disease, and largely preserved cognitive function [32,42,52]. NGS-based cohort studies have demonstrated that ML III α/β is caused by hypomorphic variants in *GNPTAB*, whereas ML III γ is associated with pathogenic variants in *GNPTG*—both subtypes exhibiting similar phenotypes, despite distinct genetic etiologies [53]. Therefore, ML II and ML III α/β, or ML III γ represent a biochemical continuum defined by residual GlcNAc-1-PTase activity. Severe truncating variants typically abolish enzyme function and result in ML II, whereas moderate to hypomorphic variants that preserve partial enzyme activity lead to ML III, producing a spectrum of phenotypes that correlate with the degree of residual enzyme activity [35].

#### 2.3.2. Pathogenic Variants in *GNPTG*

Pathogenic variants in *GNPTG*, responsible for ML III γ, typically include combinations of splice-site and truncating alleles. An uncommon early-onset ML III γ phenotype has been associated with compound heterozygous *GNPTG* variants comprising a canonical splice mutation (c.110+1G>A) and a frameshift deletion p.Asp219fsTer9, presenting with early hand stiffness and progressive joint involvement that can mimic rheumatologic disease, thereby expanding the recognized phenotypic spectrum of ML III γ [52]. Missense variants that permit partial assembly of the GlcNAc-1-PTase complex are preferentially associated with ML III rather than ML II. Examples include *GNPTAB* p.Arg587Pro and *GNPTG* p.Cys142Arg, which disrupt protein function without complete loss of enzymatic activity and are consistently linked to milder, non-neuronopathic phenotypes [54].

Genome-wide approaches have also enabled the detection of rare and complex variants not identifiable by earlier methods, with curated pathogenic variants deposited in ClinVar (ClinVar_GNPTG) Recent studies suggest that NGS-based newborn screening, combined with biochemical assays such as elevated circulating lysosomal enzyme activity, may improve early recognition of ML II or III in presymptomatic or atypical cases, supporting the feasibility of population-based newborn screening [42,55]. Collectively, molecular data support re-conceptualizing mucolipidoses II and III as a continuous spectrum of gene-variant-defined disorders rather than vaguely separated clinical entities, facilitating accurate diagnosis, prognostication, and future therapeutic stratification [35].

### 2.4. Mucolipidosis IV (ICD-11: 5C71.2)

#### 2.4.1. Pathophysiology and Clinical Presentation

ML IV is genetically and mechanistically distinct from ML II/III. It results from pathogenic variants in the *MCOLN1* gene, encoding the mucolipin-1 protein (TRPML1), a lysosomal cation channel involved in calcium release, regulation of membrane trafficking, and autophagosome-lysosome fusion (Figure 1) [56,57]. Loss of TRPML1 disrupts autophagic flux, causes accumulation of lipofuscin and phospholipids, and selectively impairs neuronal and retinal cell survival. Clinically, ML IV presents in infancy with hypotonia, global psychomotor delay, corneal clouding, retinal degeneration, achlorhydria with secondary iron-deficiency anemia, and progressive visual impairment [15]. Neuroimaging in ML IV typically reveals corpus callosum hypoplasia, diffuse white-matter attenuation, and cerebellar atrophy [58].

#### 2.4.2. Pathogenic Variants in *MCOLN1*

Pathogenicity of *MCOLN1* variants varies widely ranging from hypomorphic variants that preserve up to 40% of channel function and correlate with milder or slowly progressive phenotypes to severe deficiency and null variants. Some variants are particularly prevalent in Ashkenazi Jewish or Middle-Eastern populations and frequently diagnosed by NGS in clinical laboratories, including c.237+5G>A, p.Thr232Pro, p.Phe465Leu, p.Arg322Ter, p.Phe408del [59,60]. Dozens of canonical splice-site variants have recently been identified by WES in individuals with ML IV (e.g., c.405+1G>A, c.406-2A>G, c.680+1G>A), as well as biallelic pathogenic missense variants such as p.Tyr436His, p.Gly319Asp, p.Asp362Tyr, p.Thr121Met (ClinVar_MCOLN1) [15,61,62,63]. Moreover, heterozygous pathogenic variants in *MCOLN1* have recently been associated with Lisch epithelial corneal dystrophy (LECD) through studying multiple affected families carrying pathogenic nonsense variants p.Arg172Ter and p.Cys192Ter over several generations [64]. LECD is widely underdiagnosed, and establishing a definitive genetic diagnosis is essential. Novel gene therapy approaches to treat MCOLN1-related pathologies with systemic delivery of a newer-generation adeno-associated virus vectors (AAV-CPP16) are currently under development [65].

Meta-analyses of NGS studies and natural history studies across mucolipidoses demonstrate distinctive genotype–phenotype correlations: severe loss of lysosomal enzyme or trafficking function correlates with early-onset and increased mortality (as in classic ML II), while partial residual protein activity is associated with attenuated forms and patient survival into adolescence or adulthood (described in ML III) [16,24,66]. Similar phenotype gradients are reported in ML I cohorts with *NEU1* missense variants linked to milder late-onset disease, and ML IV patient series that document variable neurological progression over the lifespan in MCOLN1-related diseases.

## 3. Other Lysosome-Related Organelle Disorders, Including Lysosomal Membrane or Transport

Lysosomes do not function in isolation. They operate within an extended family of specialized vesicular compartments collectively termed LROs. Although LROs share many molecular features with lysosomes, they are structurally and functionally adapted to carry out distinct physiological roles: melanosomes regulate pigmentation, platelet dense granules facilitate hemostasis, and lamellar bodies manage pulmonary surfactant homeostasis [67,68]. When any component of the trafficking machinery or membrane apparatus necessary for LRO function is disrupted, highly tissue-specific disorders arise that blur the boundaries between classic LDs and defects of organelle biogenesis. NGS has been instrumental in elucidating these conditions [69]. Variants in LRO-associated genes often manifest with subtle or pleiotropic phenotypes, making unbiased genome-wide approaches essential for distinguishing pathogenic variants from benign polymorphisms. Comprehensive genomic characterization of these networks also enhances clinical diagnostic accuracy for all LDs by providing well-curated variant reference sets, thereby reducing misclassification in clinical variant interpretation pipelines.

### 3.1. Pathogenic Variants in CTNS Are Causative for Cystinosis (ICD-11: 5C74.0)

#### 3.1.1. Pathophysiology and Clinical Presentation

Cystinosis is the prototype disorder of lysosomal membrane transport. The *CTNS* gene encodes cystinosin, a proton-driven lysosomal exporter that translocates cystine from the lysosomal lumen into the cytoplasm (Figure 1). Loss or dysfunction of cystinosin results in progressive intra-lysosomal accumulation of cystine and formation of crystals, triggering cellular injury across multiple tissues [70]. Several types of cystinosis have been described (see Table 1) [71,72].

Because cystinosis has early-onset and effective targeted therapy options, it is a strong candidate for newborn genomic screening. WES and WGS performed from dried blood spots have demonstrated feasibility for early identification and genotype-guided management.

#### 3.1.2. Pathogenic Variants in *CTNS*

More than 150 pathogenic variants have been identified in the *CTNS*, including missense, nonsense, frameshift, splice-site, and large structural variants. NGS-based cohort studies demonstrate a strong correlation between variant class, predicted impact on cystinosin function, and clinical severity, making cystinosis one of the best-characterized lysosomal membrane transport disorders in terms of genotype–phenotype relationships.

Missense variants are frequently associated with residual cystinosin activity and milder or atypical presentations, such as juvenile or ocular cystinosis, although clinical expression depends on the allelic configuration. Recurrent and novel missense alleles include p.Gly308Arg, p.Leu158Pro, p.Gly339Arg, p.Gln88Lys, p.Ser139Tyr, and p.Ser141Phe [72,73,74,75]. Variants such as p.Tyr173Cys have been repeatedly associated with later-onset disease, particularly when paired with another hypomorphic allele [76]. These variants preserve partial transporter function, and generally correlate with slower renal progression and delayed extrarenal manifestations, although protein domain involvement and allelic combination may influence severity.

Truncating variants, including frameshift and nonsense mutations, are strongly associated with classic infantile nephropathic cystinosis due to near-complete loss of cystinosin function. Examples include p.Thr334Profs65, p.Gly258SerfsTer30, p.Gly258SerfsTer30, p.Gln108ArgfsTer10, p.Ser86Phefs38, and p.Thr7Phefs7 [73,74,77]. These alleles result in early-onset renal Fanconi syndrome and rapid progression to end-stage kidney disease. Similarly, nonsense variants introduce premature termination codons, phenocopying null alleles. Pathogenic splice-site variants, such as c.681+7delC and c.971-1G>C, disrupt normal transcript processing and are generally associated with moderate-to-severe disease depending on the extent of residual protein production [73]. Long-read NGS and WGS have been particularly informative in resolving complex *CTNS* structural variants, including the recurrent ~57 kb deletion spanning exons 1–10, a 20.3 kb deletion, several >1 kb insertions, and repetitive-region breakpoint rearrangements, which are not reliably detected by short-read sequencing [78,79]. Such large structural variants are consistently associated with severe infantile phenotypes. Notably, a recent clinical trial of an investigational gene therapy (self-inactivating lentiviral vector) included five patients carrying the recurrent ~57 kb deletion either in homozygous form or in compound heterozygosity with a second pathogenic *CTNS* variant [80]. Computational analyses integrating NGS datasets have additionally predicted 19 novel missense variants with high pathogenicity scores, expanding the candidate mutational spectrum and highlighting the need for functional validation to refine genotype–phenotype correlations [81]. Lifelong cysteamine therapy remains the standard of care for cystinosis. By converting lysosomal cystine into a mixed disulfide, capable of exiting the lysosome via alternative transport pathways, cysteamine significantly slows renal deterioration and improves growth when initiated early. NGS-based studies indicate that residual cystinosin activity exceeding ~40%, often seen in individuals with missense or hypomorphic splice variants, predicts improved long-term survival, preserved motor function, and delayed extrarenal complications [72,82]. Finally, NGS-based carrier screening and genetic counseling remain best practices for disease prevention in high-risk populations, enabling informed reproductive decision-making and reduction in disease incidence [83].

### 3.2. Pathogenic Variants in SCARB2 Are Causative for Action Myoclonus Renal Failure Syndrome (ICD-11: 5C74.4)

#### 3.2.1. Pathophysiology and Clinical Presentation

*SCARB2* encodes lysosomal integral membrane protein type 2 (LIMP2), a pivotal receptor mediating the trafficking of β-glucocerebrosidase (GBA) from the Golgi to lysosomes, where the enzyme is required for sphingolipid degradation (Figure 1). The enzyme β-glucocerebrosidase (GCase) depends on LIMP2 for lysosomal trafficking [84]. When LIMP2 is defective or null, GBA fails to reach its destination, impairing lipid catabolism within neurons and renal proximal tubular cells, which causes lysosomal dysfunction, membrane instability (or even lysosomal membrane rupture, as was shown in a *Caenorhabditis elegans* model) [85]. Biallelic loss-of-function variants in SCARB2 result in Action Myoclonus–Renal Failure (AMRF) syndrome, the classical phenotype. It presents in late adolescence with progressive myoclonic epilepsy, ataxia, sensorineural hearing loss, and renal failure due to glomerulosclerosis [86].

#### 3.2.2. Pathogenic Variants in *SCARB2*

Biallelic *SCARB2* variants (predominantly missense and frameshifts) have been reported by NGS, including truncating and splice variants like p.Trp178Ter, c.1239+1G>T, c.1116-2A>C, c.995-1G>A, c.435_436insAG, p.Gln288Ter, p.Glu420ArgfsTer5, p.Tyr222Ter, p.Leu14ProfsTer35, p.Asn45MetfsTer?, c.1187+5G>T, etc., which are associated with phenotypic variability in the severity and progression of neurological and renal manifestations (ClinVar_SCARB2) [87,88,89]. The above-mentioned mutations were often reported in several generations of consanguineous families with varying degrees of neuropathy, seizures, and renal involvement [90].

Multiple recent NGS reports have expanded the variant catalogue with greater than 50 new alleles, strengthening not only the *SCARB2*-AMRF gene disease relationship, but also an intricate GBA molecular crosstalk within Gaucher and Parkinson’s disease pathogeneses [91]. Heterozygous *SCARB2* variants may lead to haploinsufficiency of LIMP2, with consequent impairment of GBA trafficking and a possible contribution to susceptibility to Parkinson’s disease, although more studies are required to confirm this [92]. Miglustat, an inhibitor of glucosylceramide synthase, has been used in some patients, with mild improvement of symptoms [86].

### 3.3. Pathogenic Variants in SLC17A5 Are Causative for Sialic Acid Storage Disease (SASD) (ICD-11: 5C74.6)

#### 3.3.1. Pathophysiology and Clinical Presentation

The *SLC17A5* gene encodes solute carrier family 17 member 5 (sialin), a lysosomal membrane transporter responsible for exporting free sialic acid, predominantly N-acetylneuraminic acid (Neu5Ac), from the lysosomal lumen into the cytoplasm following degradation of glycoproteins and glycolipids (Figure 1) [93]. Sialin functions as a proton-coupled transporter, and loss of its activity disrupts the normal efflux of sialic acid from lysosomes, leading to its intracellular accumulation and impairment of cellular homeostasis, particularly in neurons [93]. Deficiency of sialin causes SASD, a spectrum of autosomal recessive lysosomal disorders that includes a severe infantile form (infantile free sialic acid storage disease, IFSD) and a milder, later-onset phenotype historically referred to as Salla disease. These are now generally considered part of a continuous clinical spectrum rather than distinct entities [94]. In sialin-deficient cells, free sialic acid accumulates within lysosomes, leading to progressive cellular dysfunction, particularly affecting the central nervous system.

The severe infantile form typically presents in early infancy with developmental delay, coarse facial features, hepatosplenomegaly, and marked hypotonia, followed by rapid neurological deterioration. In contrast, milder SASD phenotypes present with early hypotonia and delayed motor development, followed by progressive neurological manifestations such as ataxia, nystagmus, and, in some cases, epilepsy. Neuroimaging commonly demonstrates hypomyelination and cerebral and cerebellar atrophy. Affected individuals often develop persistent ataxia and varying degrees of intellectual disability [95].

Biochemical confirmation may involve detection of elevated free sialic acid levels in urine or cultured cells; however, such specialized assays are not widely available in routine clinical laboratories [96]. Consequently, next-generation sequencing (NGS) has become the primary diagnostic approach for confirming SLC17A5-related disease, particularly in individuals presenting with nonspecific neurodevelopmental phenotypes.

#### 3.3.2. Pathogenic Variants in *SLC17A5*

The *SLC17A5* gene demonstrates substantial allelic heterogeneity, with reported pathogenic variants including missense substitutions, nonsense (truncating) variants, frameshift mutations, splice-site alterations, and larger structural variants detectable by genomic sequencing approaches. Data from ClinVar indicate that loss-of-function variants—particularly nonsense and frameshift mutations—are prominent, alongside copy number variants such as deletions and duplications, although the exact distribution may be influenced by reporting bias (ClinVar_SLC17A5). Representative truncating variants include p.Glu15Ter, p.Trp246Ter, p.Leu193fs, p.Tyr223fs, and p.Met1Ter. The missense variant p.Arg39Cys is a well-established founder mutation, particularly enriched in Finnish populations, and is strongly associated with the milder end of the SASD spectrum. Additional missense variants, such as p.Gly313Ala and p.Gly328Glu, have been identified through NGS studies and are predicted to affect functionally important luminal regions of sialin [94]. WGS has also revealed complex mutational mechanisms. For example, Tarailo-Graovac et al. described a homozygous ~6 kb LINE-1 retrotransposon insertion in intron 9 of *SLC17A5*, which introduces cryptic splice sites and leads to aberrant splicing of the transcript. This splicing defect results in the incorporation of intronic sequence, causing a frameshift and subsequent premature termination of the encoded protein [95]. These findings highlight the importance of approaches capable of detecting structural and noncoding variants. Because SASD shares overlapping clinical features with other lysosomal disorders, inclusion of *SLC17A5* in targeted NGS panels is essential for timely diagnosis and for avoiding misclassification with phenotypically similar conditions.

At present, treatment for free sialic acid storage disease remains largely supportive. Due to the absence of disease-modifying therapies, the disorder does not currently meet the primary criteria for inclusion in the Recommended Uniform Screening Panel (RUSP) for newborn screening in the United States. Therefore, NGS-based carrier screening and molecular diagnosis remain the most effective strategies for identifying affected individuals and preventing recurrence within at-risk families.

### 3.4. Genes Affected in Hermansky–Pudlak Syndrome (HPS) (ICD-11: 5C74.8)

**Figure 2:**
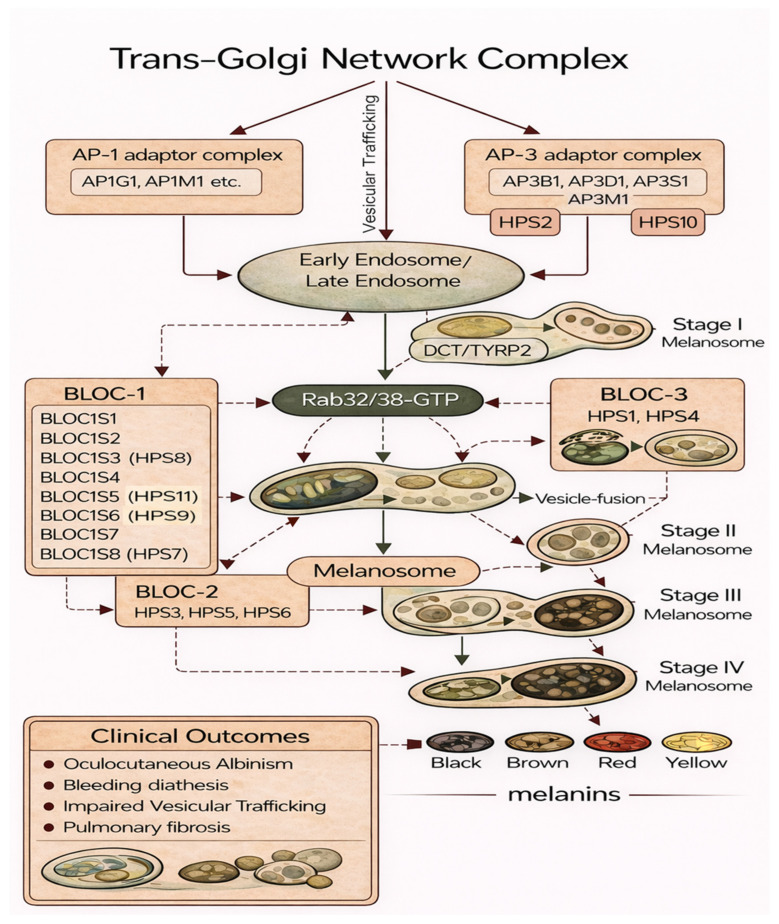
Simplified diagram of the role of BLOC complexes in endosome and melanosome biogenesis. Dashed arrows represent interaction of BLOC complexes with endosome or melanosome. HPS types are depicted next to corresponding defective LRO genes. This figure is designed with ChatGPT 5.0.

HPS subtypes comprise a group of autosomal recessive disorders caused by defective biogenesis and trafficking of LROs, including melanosomes, platelet dense granules, and lamellar bodies (Figure 2). The clinical phenotype typically begins in infancy with hypopigmentation and bleeding manifestations, while additional systemic complications may develop later in childhood or adulthood. Platelets from individuals with HPS show a characteristic defect in the secretion of dense (δ)-granules, reflecting the absence or dysfunction of platelet dense (δ)-granules, which are CD63-positive organelles. Various molecular defects disrupt intracellular trafficking pathways mediated by the biogenesis of lysosome-related organelles complexes (BLOC-1, BLOC-2, and BLOC-3) and the adaptor protein complex AP-3. Impaired function of these complexes alters vesicular transport and cargo delivery to specialized organelles, resulting in the hallmark clinical features of oculocutaneous albinism and bleeding diathesis [97].

Additional complications, including progressive pulmonary fibrosis, granulomatous colitis, reduced visual acuity, immunodeficiency, neurodevelopmental abnormalities, and occasionally, impaired renal function, are increasingly recognized as genotype-dependent and subtype-specific, as described below. In particular, pulmonary fibrosis represents a major source of morbidity and mortality and occurs predominantly in HPS1, HPS2, and HPS4, whereas other subtypes more commonly exhibit milder phenotypes without significant pulmonary involvement [98,99]. Furthermore, the distribution of HPS subtypes reflects population-specific genetic architectures, including allelic heterogeneity and founder effects, resulting in distinct mutational spectra across different ethnic groups, for example, the high prevalence of HPS1 in Puerto Rican populations and the predominance of *HPS1* and *HPS6* variants, including multiple novel alleles, in East Asian cohorts such as Chinese patients [100,101].

The BLOC complexes play a central role in the biogenesis and maintenance of LROs, and pathogenic variants in genes encoding their subunits define distinct HPS subtypes. These complexes operate within a broader network of lysosomal trafficking regulators, including proteins associated with the BLOC-one-related complex (BORC), which governs lysosome positioning and membrane dynamics. Functional and structural overlap among these pathways contributes to partial phenotypic convergence across related trafficking disorders [102]. A key challenge in clinical genomics remains distinguishing pathogenic variants from benign findings in HPS-associated genes.

Recent advances in structural biology, including cryo-electron microscopy, have begun to resolve the architecture of BLOC complexes, providing insights into their assembly and interactions (Figure 2). Integration of structural data with genomic findings from patient sequencing allows prediction of how specific mutations disrupt protein binding, subcellular localization, and complex stability [103]. Complementary functional studies using cellular and in vitro models have further defined the coordinated roles of BLOC-1, BLOC-2, BLOC-3, AP-3, and the homotypic fusion and protein sorting complex in regulating vesicle formation, targeting, and fusion during LRO maturation. Together, these approaches have significantly advanced understanding of HPS molecular pathogenesis and genotype–phenotype correlations [104,105].

#### 3.4.1. Pathogenic Variants in HPS1

Pathogenic variants in *HPS1*, encoding a subunit of the BLOC-3 complex, represent the most common cause of HPS worldwide and account for the largest number of pathogenic variants identified to date. Loss-of-function variants impair endosomal trafficking pathways required for melanosome maturation and platelet dense granule biogenesis (Figure 2). Clinically, individuals affected with HPS type 1 present with severe hypopigmentation and bleeding diathesis, together with a high risk of progressive pulmonary fibrosis and impaired renal function that usually manifests in adulthood [106,107].

Since the introduction of NGS, numerous additional pathogenic variants have been identified globally, including several founder alleles, and both the protein structure and the distribution of *HPS1* variants are now well described (ClinVar_HPS1) [108]. These include primarily frameshift variants, as well as nonsense and missense variants: p.Met325HisfsTer128, p.Trp146Ter, p.Met325TrpfsTer6, p.Cys3TrpfsTer26, p.Arg493GlyfsTer22, p.Asp292AlafsTer38, p.Asp427GlyfsTer27, p.Gln686Ter, p.Glu616Lys, p.Leu585Pro [109,110,111,112,113]. The availability of NGS-based diagnostic approaches has enabled large cohort studies and longitudinal analyses, improving differential diagnosis, characterization of mutational spectra, and assessment of pulmonary fibrosis-related morbidity and mortality [114,115,116]. Advanced sequencing strategies have revealed pathogenic mechanisms that are difficult to detect using DNA-based approaches alone. While WGS can identify deep intronic variants, their impact on splicing is often uncertain, as they lie outside canonical splice sites and are not reliably predicted by in silico tools. RNA-seq allows direct evaluation of mRNA transcript structure and splicing alterations. In HPS1, this combined approach identified a deep intronic variant, c.1397+135C>T, which was not predicted to affect splicing but was shown by RNA-seq to activate a pseudoexon, leading to aberrant transcript inclusion and a premature termination codon. These findings underscore the value of RNA-seq in confirming the functional effects of noncoding variants that may remain unresolved by DNA-based analyses alone [117]. Such findings illustrate the importance of integrated genomic and transcriptomic approaches for identifying noncoding variants that disrupt gene expression. Recent studies further suggest that disease progression in HPS1 may involve inflammatory and immune-mediated mechanisms. A regulatory network associated with age-related neutrophil activation, characterized by increased inflammatory cytokines, neutrophil granule proteins, and enhanced neutrophil extracellular trap formation, has been linked to the development and progression of pulmonary fibrosis in individuals with pathogenic variants in HPS1 [118].

Because albinism can result from mutations in genes beyond those associated with HPS, differential diagnosis using NGS is essential [111]. In some reported cases, an initial clinical suspicion of HPS1-related disease was later revised following genetic testing that identified variants in other pigmentation genes. For instance, four hypomorphic variants in the tyrosinase gene (*TYR*) were detected in a patient initially suspected of having HPS1, leading to a final diagnosis of oculocutaneous albinism type 1B [119].

#### 3.4.2. Pathogenic Variants in *AP3B1*

Pathogenic variants in *AP3B1* cause HPS type 2 by disrupting the AP-3, which mediates the sorting of proteins destined for lysosomes and lysosome-related organelles (Figure 2). In addition to oculocutaneous albinism and platelet dense (δ)-granule deficiency, AP3B1 deficiency is distinguished by immunodeficiency, chronic neutropenia, platelet dysfunction, and ceroid deposition. The disorder therefore combines defects in pigmentation, hemostasis, and immune function. Clinical presentation associated with pathogenic variants in *AP3B1* shows variable onset and expressivity. Reported manifestations range from neonatal interstitial lung disease to recurrent infections during childhood and periodontal inflammation in adolescence, reflecting impaired vesicular trafficking and granule formation in immune cells [120,121].

Multiple compound heterozygous variants have been reported in patients, including nonsense, frameshift, splice-site variants, and large deletions that lead to severe reduction or absence of AP-3 complex function (ClinVar_AP3B1). Examples include c.[177delA];[1839-1842delTAGA], p.[Gln255 Leu];[Glu19AspfsTer21], p.[Met63Lys];[Leu849Ter], p.[Phe375SerfsTer11];[p.Leu849Ter], and the compound heterozygotes c.205-1G>C and p.Asn4LysfsTer6 [120,121,122,123]. Functional studies confirmed that these variants disrupt lysosomal targeting and trafficking of granule proteins, providing a mechanistic explanation for the combined hematologic and immunologic phenotype. Several consanguineous multigenerational families have also been described carrying homozygous deletions, missense or truncating variants, including p.Val128Ala, p.Val640GlyfsTer29, p.Glu693fsTer13, p.Glu52fsTer11 [124,125].

Beyond classical HPS2, heterozygous variants in *AP3B1* have also been associated with immune dysregulation. Germline variants in *AP3B1* and *UNC13D* were reported to be enriched in patients with severe COVID-19-associated cytokine storm, suggesting that defects in cytotoxic granule trafficking pathways may contribute to hyperinflammatory responses [126]. Gao et al. described an adult patient with hemophagocytic lymphohistiocytosis (HLH) carrying heterozygous variants in both *UNC13D* (c.1232G>A) and *AP3B1* (c.1075A>G), supporting a synergistic effect of double heterozygosity in genes involved in lymphocyte cytotoxicity and vesicle trafficking [127]. Similarly, Yin et al. reported a pediatric HLH case with heterozygous variants in *AP3B1* (c.3197C>T) and *ATM* (c.8077G>T), presenting with severe cytokine storm, distributive shock, and multiorgan dysfunction syndrome, further implicating *AP3B1* as a contributor to digenic immune dysregulation [128].

#### 3.4.3. Pathogenic Variants in HPS3

Pathogenic variants in *HPS3*, which encodes a component of the BLOC-2 complex, cause a relatively mild HPS subtype (HPS type 3) (Figure 2). Affected individuals typically show moderate hypopigmentation and a bleeding tendency, without the severe systemic complications, particularly pulmonary fibrosis, seen in other subtypes. The phenotype includes oculocutaneous albinism, reduced visual acuity, nystagmus (sometimes with compensatory head movements), and occasional gastrointestinal involvement such as enterocolitis. Immune dysfunction is not a consistent feature, further supporting the mild systemic nature of this subtype.

*HPS3* is notable for the presence of recurrent founder variants in certain populations, contributing to increased prevalence in specific geographic and ancestral groups [129]. Expanded NGS studies have demonstrated that residual BLOC-2 activity correlates with reduced systemic manifestations, providing a mechanistic explanation for the milder phenotype observed in many affected individuals. However, recent reports describing gastrointestinal inflammation and inflammatory bowel disease-like features suggest broader phenotypic variability than previously recognized [130]. High-throughput sequencing approaches have identified a wide spectrum of pathogenic variants in *HPS3*, including missense, nonsense, frameshift, splice-site variants, and large deletions (ClinVar_HPS3). Recurrently reported variants include c.1163+1G>A, p.Pro22Arg, p.Cys398Tyr, p.Tyr922Ter, p.Arg822Ter, p.Gln3Ter, p.Asp411GlyfsTer32, p.Ser613Ter, p.Leu333Ter, p.Glu963Ter, and p.Leu533PhefsTer10 [112,129,131,132,133,134,135,136,137]. In addition, a 14,761-bp pathogenic deletion involving the 5′ untranslated region has been reported, further expanding the mutational landscape of *HPS3* [135]. The persistence of *HPS3* founder variants (like p.Ser613Ter in the Chinese population, and c.1163+1G>A, c.2621-2A>G, c.1831+2T>G in the Ashkenazi Jewish population) underscores the role of population history in shaping the distribution of rare disease alleles, and highlights the importance of ancestry-informed NGS diagnostics and community-engaged genomic medicine [135,138].

#### 3.4.4. Pathogenic Variants in *HPS4*

Pathogenic variants in *HPS4*, which encodes the second subunit of the BLOC-3 complex, cause a clinical phenotype that is largely indistinguishable from HPS1-related disease (Figure 2). Individuals affected with HPS type 4 typically present with oculocutaneous albinism, platelet dense granule deficiency, and a high risk of progressive pulmonary fibrosis and granulomatous colitis. Early mutation studies identified truncating and missense variants in diverse populations, establishing HPS4 as a major non-Puerto Rican cause of severe Hermansky-Pudlak syndrome [139]. Functional analyses demonstrated that the integrity of the BLOC-3 complex is essential for late endosomal trafficking and lysosome-related organelle biogenesis. Multiple patients have been reported carrying homozygous pathogenic variants in *HPS4* (in familial cases) that result in frameshift, nonsense, splice-site, or missense alterations (ClinVar_HPS4). Reported variants include p.Pro685LeufsTer17, p.Val394Pro395fsTer23, p.Ser280ProfsTer34, c.1713+1delG, p.Leu91Pro, p.Ala211fsTer, and p.Trp139Ter [112,131,134,137,140,141]. These variants disrupt BLOC-3 complex stability and impair vesicular trafficking pathways required for proper maturation of lysosome-related organelles.

Recent genomic and transcriptomic studies have also identified *HPS4* as a lysosome-related gene with potential biomarker value. Experimental knockdown of HPS4 in liver cancer cell models suppressed tumor cell proliferation and induced apoptosis. Prognostic prediction models incorporating *HPS4* expression further highlighted its potential value as a biomarker and therapeutic target in hepatocellular carcinoma [142]. These findings suggest that regulation of *HPS4* may provide novel strategies for precision treatment in liver cancer, and further investigation of HPS4-associated pathways may also provide insights into lysosome-related mechanisms in non-germline disease contexts.

#### 3.4.5. Pathogenic Variants in *HPS5*

Pathogenic variants in *HPS5*, which encodes a subunit of the BLOC-2 complex, cause HPS type 5 (Figure 2). HPS5 associates with HPS3 as part of the biogenesis of lysosome-related organelles complex-2 (BLOC-2), which is required for trafficking processes involved in lysosome-related organelle formation. Identified pathogenic variants include missense and truncating alleles that lead to partial destabilization of the BLOC-2 complex (Figure 2). Clinical series and case reports indicate a low incidence of pulmonary and gastrointestinal complications as compared with BLOC-3-related disease, and many patients present with mild hypopigmentation and bleeding tendency without additional major complications [143]. Reported variants include homozygous p.Val254Phe and compound heterozygous variants, such as p.[Glu634Ter];[Trp246Ter] and p.[Gly50Asp];[Cys360Arg] (ClinVar_HPS5) [133,144]. In some cases, patients exhibit mild bleeding symptoms that do not substantially affect quality of life and maintain a normal life span. For example, individual carrying the homozygous p.Lys654Ter variant who was diagnosed with platelet dense granule storage pool deficiency (DG-SPD) at the age of 38 years, after presenting with spontaneous intracranial hemorrhage [145]. During the subsequent two decades of follow-up the patient did not develop severe bleeding, pulmonary or gastrointestinal complications.

Complex genetic findings have also been described. A combination of a heterozygous variant of uncertain significance in *HPS5* (p.Tyr956His) together with a homozygous nonsense variant (p.Arg47Ter) in the *HPS6* gene was reported in a 24-year-old patient with renal failure and an atypical HPS presentation, suggesting potential interactions between components of the BLOC-2 complex and interconnected molecular mechanisms [146].

#### 3.4.6. Pathogenic Variants in *HPS6*

Pathogenic variants in *HPS6*, another BLOC-2 component, result in HPS type 6, with a phenotype similar to HPS type 5. HPS6 is required for proper trafficking to lysosome-related organelles, particularly melanosomes and platelet dense granules (Figure 2). Functional studies demonstrate defective melanosome maturation and impaired platelet dense granule formation, underlying the characteristic hypopigmentation and bleeding diathesis. While lysosome-related organelles such as Weibel-Palade bodies (WPBs) in endothelial cells share related biogenesis pathways, the specific role of HPS6 in their maturation remains less well defined. Additionally, HPS6 is likely to be involved in the maturation of WPB, which is one of the LROs in endothelial cells, whose primary cargo is von Willebrand factor (vWF) [104]. HPS6 deficiency has been reported to result in misshapen, immature WPBs and reduced storage of vWF, impairing primary hemostasis and contributing to bleeding disorders.

Pathogenic *HPS6* variants classified as PVS1 (ACMG criteria [10,146]) are typically loss-of-function alleles, including p.Arg667Ter, p.Trp112Ter, p.Arg578Ter, p.Gln341Ter, p.Gly382GlyfsTer13, p.Asn559fsTer, p.Asn559GlnfsTer8, p.Gly519ValfsTer1, and p.Leu22ArgfsTer33 [109,133,134,147,148]. Some variants have been observed in a double homozygous state, such as c.1789delG, p.Ser379Ter, and p.Ala597GlnfsTer16, in members of consanguineous families presenting with severe oculocutaneous albinism [149]. Recently, novel homozygous missense variants in HPS6 (p.Val128Ala, p.Ala81Ser, p.Leu378Arg) were reported to markedly reduce HPS6 mRNA or protein expression (ClinVar_HPS6). These variants correlated with the absence of platelet δ-granules on whole-mount electron microscopy, confirming a significant platelet storage pool defect despite a clinically mild HPS phenotype [117,150].

A very rare and unusual case combining hemophilia B and HPS6 was reported, demonstrating the absence of platelet δ-granules on electron microscopy, oculocutaneous albinism, abnormal secondary wave in platelet aggregation studies, and a homozygous p.Arg372Ter variant [151]. This case highlights the potential for atypical presentations and overlapping hematologic disorders in patients with *HPS6* pathogenic variants.

#### 3.4.7. Pathogenic Variants in *DTNBP1*

Pathogenic variants in *DTNBP1* (also known as *BLOC1S8,* OMIM: 607145), which encodes the protein dysbindin, a component of the BLOC-1 complex, cause HPS type 7 (Figure 2). This subtype is extremely rare and remains poorly characterized due to the limited number of reported cases. Affected individuals typically present with oculocutaneous albinism and bleeding diathesis. Reported pathogenic variants include frameshift and nonsense alleles that result in complete loss of dysbindin expression, often associated with an early-onset inflammatory bowel disease [152]. NGS studies have confirmed these variants, with the nonsense variant p.Gln103Ter in *DTNBP1* being the one of the most frequently reported [134,152,153]. A complex homozygous deletion of exon 6 in *DTNBP1* c.(355+1_356-1)_(488+1_489-1)del resulted in complete loss of dysbindin expression and additional phenotypic features, including recurrent bacterial infections and reduced natural killer (NK) cell degranulation [144]. To date, over 30 pathogenic or likely pathogenic variants in *DTNBP1* have been reported in ClinVar (DTNBP1_ClinVar). The rarity of these cases underscores the ultra-rare status of HPS7 and highlights the need for continued genomic studies to define the full spectrum of clinical and molecular features associated with this subtype.

#### 3.4.8. Pathogenic Variants in *BLOC1S3*

Pathogenic variants in *BLOC1S3* cause HPS type 8 (HPS8) by disrupting a subunit of the biogenesis of lysosome-related organelles complex 1 (BLOC-1) (Figure 2). Reported patients exhibit classic HPS features of moderate severity. At least four consanguineous families with HPS8 have been described, each carrying distinct homozygous pathogenic variants in *BLOC1S3*, including c.385_403del, c.338_341del, c.444_467del, and p.Gln150ArgfsTer75. Affected individuals consistently present with moderate oculocutaneous albinism and bleeding diathesis. Additional ocular manifestations reported include nystagmus, iris transillumination, foveal hypoplasia, reduced visual acuity, and evidence of optic pathway misrouting [154,155]. Rare additional complications have also been described. For example, one reported patient with HPS8 developed lymphocyte-predominant Hodgkin lymphoma [155]. 

Aside from these cases, very limited information is available regarding the pathogenicity of variants in *BLOC1S3*. In ClinVar, only four pathogenic variants have been submitted in association with HPS8, including p.Gln150_Ala157del, p.Ser129fs, p.Leu113fs, and p.Ser44fs, and *BLOC1S3* has also been reported within large multigenic copy number gains on chromosome 19q (ClinVar_BLOC1S3).

#### 3.4.9. Pathogenic Variants in *BLOC1S6*

Pathogenic variants in *BLOC1S6* (also known as *PLDN*) were initially identified through candidate gene sequencing and functional studies demonstrating impaired BLOC-1 complex stability (Figure 2). Individuals with HPS type 9 (HPS9) present with oculocutaneous albinism and platelet dense granule deficiency. The rarity of reported cases suggests that the condition is underdiagnosed; this limitation is increasingly addressed through NGS-based gene panels, WES, and WGS (ClinVar_BLOC1S6). Novel compound heterozygous variants have been reported, including p.[Glu50Ter];[Ile118TyrfsTer10] and p.[Ser67Ter];[Glu107Met], identified by whole-exome sequencing [156,157]. Homozygous variants are predominantly observed in consanguineous families, such as a deletion of exon 1 in *BLOC1S6*, p.His96LeufsTer22, and c.224+1G>A [117,158,159].

A number of common heterozygous variants in *BLOC1S6* have been reported in genome-wide association studies (GWAS) and appear to modulate melanin levels and pigmentation in various populations. These variants may contribute to pigmentation differences in the general population without necessarily causing HPS [160].

#### 3.4.10. Pathogenic Variants in *AP3D1*

Pathogenic variants in *AP3D1*, which encodes the δ subunit of the AP-3 complex, cause HPS type 10 (HPS10) (Figure 2), a syndromic form characterized by oculocutaneous albinism, bleeding diathesis, immunodeficiency, increased susceptibility to infection, neutropenia, seizures, and severe neurodevelopmental delay [161]. Homozygous truncating variants have been reported, with functional studies demonstrating global AP-3 dysfunction affecting both neuronal and immune pathways (ClinVar_AP3D1). Examples of such pathogenic variants include p.Val1189LeufsTer8, c.1859+1G>T, and p.Ala660ArgfsTer54 [162,163,164]. In addition, homozygous pathogenic missense variants, such as p.Val711Ile and p.Val1064Ile, segregate with severe phenotypes including sensorineural hearing loss, early-onset epilepsy, and early childhood mortality, suggesting that these residues may reside within functionally critical domains of AP3D1 protein [165,166].

Animal models, including zebrafish (*Danio rerio*) and murine model of HPS10, have been recently developed to study AP3D1 function and the molecular pathogenesis of this rare disease, providing platforms for mechanistic and therapeutic studies [167,168].

Aside from its role in HPS10, AP3D1 has been implicated in the regulation of interferon-γ and MHC-I signaling in cancer-related contexts. Specifically, AP3D1 facilitates the lysosomal trafficking of IFNGR1, promoting its degradation. Under normal conditions, optineurin interacts with AP3D1 and restrains this process. However, loss of optineurin in cancer enhances AP3D1-mediated lysosomal sorting of IFNGR1 and has been associated with reduced antitumor T-cell responses and resistance to immunotherapy in colorectal cancer [169]. Additionally, AP-3 complex subunit δ has been identified as an atherosclerosis-associated antigen, with elevated anti-AP3D1 serum IgG levels correlating with increased risk of acute ischemic stroke, diabetes mellitus, and chronic kidney disease. These findings suggest its potential utility as a biomarker for atherosclerotic disease progression and stroke risk [170].

#### 3.4.11. Pathogenic Variants in *BLOC1S5*

Pathogenic variants in *BLOC1S5* (formerly known as Muted) were formally recognized as a distinct HPS type (HPS11) following NGS-based discovery (ClinVar_BLOC1S5). *BLOC1S5* encodes the muted subunit of the obligate multisubunit BLOC-1 complex (Figure 2). Variants in this gene disrupt BLOC-1 assembly, leading to defective biogenesis of lysosome-related organelles. Reported patients present with typical HPS features, including oculocutaneous albinism and platelet dense granule deficiency, but without consistent pulmonary involvement [171].

Pennamen et al. screened 230 unresolved HPS patients using NGS and identified a deletion encompassing exon 3 of *BLOC1S5* (c.196-678_384+3483del) in one patient, and a homozygous p.Val116SerfsTer19 variant in another, thereby ending the diagnostic odyssey and establishing the molecular diagnosis of HPS11 in both cases [171]. Newly identified homozygous variants in *BLOC1S5*, including c.113-1G>A and p.Val61Ter, were associated with severely impaired platelet δ-granule secretion, as indicated by reduced CD63 expression [172,173].

In addition, a novel *IRF4-BLOC1S5* fusion gene was reported in a patient with TEMPI syndrome (telangiectasias, elevated erythropoietin and erythrocytosis, monoclonal gammopathy, perinephric fluid collections, and intrapulmonary shunting), raising the possibility of a digenic contribution and further expanding the spectrum of multisystem phenotypes associated with this rare disorder [174].

In summary, HPS spectrum disorders are defined by defects in lysosome-related organelle biogenesis, with hallmark features of oculocutaneous albinism, bleeding diathesis, and variable systemic involvement. Clinical presentation and severity are highly heterogeneous, reflecting differences in the affected gene, variant type, and molecular complex. Currently, no curative therapy exists for HPS, and clinical care remains supportive, focusing on symptom-directed management. This includes prevention and treatment of complications such as bleeding, inflammatory bowel disease, and pulmonary fibrosis (including lung transplantation) [175]. Ongoing research into vesicle trafficking and lysosome-related organelle biology continues to inform potential future therapeutic strategies, including gene- and molecular pathway-targeted approaches. Carrier screening in at-risk populations should be considered to prevent these debilitating disorders.

The continued application of NGS is expected to identify additional genes and pathogenic variants in HPS and related LRO disorders. Of particular interest are genes shared between early- and late- endosomal/lysosomal biogenesis, such as *BLOC1S1* (OMIM: 601444), *BLOC1S2* (OMIM: 609768), and *BLOC1S4* (OMIM: 605695), where new genotype–phenotype correlations in humans may emerge. However, some shared subunits, such as *SNAPIN* (also known as *BLOC1S7*, OMIM: 607007), have been associated with autosomal recessive neurodevelopmental disorders with structural brain and craniofacial abnormalities (OMIM: 621393), and more resemble a BORC-complex phenotype rather than classical HPS. Additionally, *CD63* (*TSPAN30*; OMIM: 155740), although not yet assigned an official HPS subtype, represents a candidate gene for emerging lysosome-related organelle disorders.

## 4. Conclusions and Future Perspectives

NGS-enabled clinical, carrier, and newborn screening have begun to incorporate molecular confirmation for selected lysosomal storage and transport disorders, highlighting the emerging role of genomics in population-level early detection [138]. Genomic testing expands diagnostic and screening opportunities but also identifies variants of uncertain significance. Strategies to mitigate the potential challenges of VUS include testing protocols that limit their identification or reporting, subclassifying VUS according to predicted pathogenicity, routine family-based variant evaluation, and enhanced genetic counseling [176].

Artificial intelligence is increasingly applied to comprehensive genome interpretation, enabling nomination of candidate diagnoses for rare genetic diseases. Analysis of previously unsolved cases using AI has led to novel findings that have resolved diagnostic odysseys [177]. Several commercial and academic genomic laboratories now offer periodic genome re-evaluation using AI-driven data mining to identify novel causative variants, further enhancing the diagnostic yield for rare lysosomal disorders.

## Figures and Tables

**Table 1:** Genotype–Phenotype Correlation of *CTNS* Variants in Cystinosis Subtypes.

Subtype	Clinical Features	Key Variant Types *	Severity
Infantile nephropathic cystinosis	Most severe form. Presents in infancy (typically 6–12 months) with Fanconi syndrome, growth retardation, and progressive proximal tubular dysfunction. Without treatment, progresses to end-stage kidney disease in childhood.	*CTNS* loss-of-function variants including large deletions, nonsense, frameshift, canonical splice-site variants, and severe missense variants affecting functionally important domains. Biallelic (homozygous or compound heterozygous).	Severe
Juvenile (intermediate) cystinosis	Later onset in childhood or adolescence with slower progression of renal dysfunction. Less aggressive than infantile form but still leads to chronic kidney disease.	Biallelic *CTNS* variants with residual protein function, commonly missense or splice-region variants, including some combinations of one severe + one milder allele. May or may not involve functionally important domains.	Moderate
Ocular (non-nephropathic) cystinosis	Primarily corneal cystine crystal deposition causing photophobia and ocular discomfort. No clinically significant renal disease.	Hypomorphic *CTNS* variants that preserve partial function; often missense variants outside key functional regions or mild regulatory/splice variants in biallelic form.	Mild

* This table was compiled based on data from (ClinVar_CTNS) and the referenced literature.

## Data Availability

This study is a review of previously published literature. No new datasets were generated or analyzed. All data supporting the findings are available in the 177 cited references.

## Abbreviations

ACMG	American College of Medical Genetics and Genomics
AMRF	Action myoclonus-renal failure syndrome
AP3B1	Adaptor-related protein complex 3 subunit beta 1
AP-3	Adaptor protein complex
AP3D1	Adaptor-related protein complex 3 subunit delta 1
BLOC-1	Biogenesis of lysosome-related organelles complex 1
BLOC1S3	Biogenesis of lysosomal organelles complex 1 subunit 3
BLOC-2	Biogenesis of lysosome-related organelles complex 2
BLOC-3	Biogenesis of lysosome-related organelles complex 3
BORC	BLOC-one-related complex
BLOC1S6	Biogenesis of lysosomal organelles complex 1 subunit 6
BLOC1S5	Biogenesis of lysosomal organelles complex 1 subunit 5
CD63	Cluster of differentiation 63
CTNS	Cystinosin, lysosomal cystine transporter
DTNBP1	Dystrobrevin binding protein 1
GNPTAB	N-acetylglucosamine-1-phosphate transferase subunits alpha and beta
GNPTG	N-acetylglucosamine-1-phosphate transferase subunit gamma
GlcNAc-1-PTase	N-acetylglucosamine-1-phosphotransferase
GBA	β-glucocerebrosidase
GCase	β-glucocerebrosidase
HPS	Hermansky–Pudlak Syndrome
HPS1	HPS1 biogenesis of lysosomal organelles complex 3 subunit 1
HPS2	Hermansky–Pudlak Syndrome type 2
HPS3	Hermansky–Pudlak Syndrome type 3
HPS4	Hermansky–Pudlak Syndrome type 4
HPS5	Hermansky–Pudlak Syndrome type 5
HPS6	Hermansky–Pudlak Syndrome type 6
HPS7	Hermansky–Pudlak Syndrome type 7
HPS8	Hermansky–Pudlak Syndrome type 8
IFSD	Infantile free sialic acid storage disease
IRF4	Interferon regulatory factor 4
LSDs	Lysosomal storage diseases
LROs	Lysosome-related organelles
LDs	Lysosomal diseases
LIMP2	Lysosomal integral membrane protein type 2
ML	Mucolipidoses
MCOLN1	Mucolipin-1
M6p	Mannose-6-phosphate
NGS	Next-generation sequencing
NEU1	Neuraminidase 1
Neu5Ac	N-acetylneuraminic acid
NK	Natural killer
RUSP	Recommended Uniform Screening Panel
RNA-seq	Ribonucleic acid sequencing
SCARB2	Scavenger Receptor Class B Member 2
SASD	Sialic Acid Storage Disease
SLC17A5	Solute Carrier Family 17 Member 5
TEMPI	Telangiectasias, elevated erythropoietin and erythrocytosis, monoclonal gammopathy, perinephric fluid collections, and intrapulmonary shunting
TREM2	Triggering Receptor Expressed on Myeloid cells 2
TRPML1	mucolipin-1 protein
TYR	Tyrosinase gene
WES	Whole-exome sequencing
WGS	Whole-genome sequencing
